# Diet and Nondiet Predictors of Urinary 3-Phenoxybenzoic Acid in NHANES 1999–2002

**DOI:** 10.1289/ehp.11082

**Published:** 2008-04-11

**Authors:** Anne M. Riederer, Scott M. Bartell, Dana B. Barr, P. Barry Ryan

**Affiliations:** 1 Department of Environmental and Occupational Health, Rollins School of Public Health, Emory University, Atlanta, Georgia, USA; 2 Program in Public Health and Department of Epidemiology, University of California, Irvine, Irvine, California, USA; 3 National Center for Environmental Health, Centers for Disease Control and Prevention, Atlanta, Georgia, USA

**Keywords:** 3-phenoxybenzoic acid, biomarkers, dietary exposure, pesticides, pyrethroids

## Abstract

**Background:**

3-Phenoxybenzoic acid (3PBA), a pyrethroid metabolite, was detected in 75% of urine samples analyzed for pesticides in the U.S. National Health and Nutrition Examination Survey (NHANES) 1999–2002. NHANES also includes 24-hr diet data and information on household pesticide use, activities, occupation, demographics, and other exposure factors.

**Objectives:**

The objective of our study was to explore the relative importance of diet versus nondiet predictors in explaining variability in urinary 3PBA. A secondary objective was to explore whether the NHANES data could be used to identify particular foods driving 3PBA levels.

**Methods:**

We divided subjects into child (6–10 years of age), teen (11–18 years), and adult (≥ 19 years) age groups and restricted our analyses to subjects in the morning sampling session who fasted for ≥ 8 hr beforehand. Regression modeling consisted of several model-building steps and a final Tobit regression on the left-censored log 3PBA measurements. We also conducted bootstrap analyses to evaluate the stability of the regression parameters.

**Results:**

Reported household pesticide use was not significantly associated with urinary 3PBA in any age group. Diet was significant for all three groups, and certain foods appeared to contribute more than others. Among adults, tobacco use was positively associated with 3PBA (*p* = 0.0326), and positive associations were suggested with the number of cytochrome p450–inhibiting medications taken (*p* = 0.0652) and minutes spent gardening (*p* = 0.0613) in the past month.

**Conclusions:**

Although exploratory, our findings underline the importance of collecting accurate data on household pesticide use and dietary intake when evaluating pyrethroid exposure–biomarker relationships.

Pyrethroids are the latest class of insecticides in global widespread use and are replacing organophosphates in agricultural and consumer applications (Nishi et al. 2006). Pyrethroids exert their neurotoxicity by slowing the opening and closing of voltage-gated sodium channels in insect and mammalian nerve cells (Shafer et al. 2005). Recent toxicologic studies show interference with chloride channels and other target systems (Ray and Fry 2006; Shafer and Meyer 2004). Although the acute toxicity to humans is well documented (Spencer and O’Malley 2006), data on health effects of lower-level exposures are currently limited to animal studies. Findings from these suggest that additional research on human exposures is warranted. In their review of 22 rodent studies, for example, Shafer et al. (2005) report associations between *in utero* pyrethroid exposures and persistent changes in neurochemistry, motor activity, behavior, and learning.

In July 2005, the U.S. Centers for Disease Control and Prevention (CDC) reported measurable levels of 3-phenoxybenzoic acid (3PBA), a metabolite of several commonly used pyrethroids, including permethrin, cypermethrin, cyhalothrin, deltamethrin, and fenvalerate, in 75% of urine samples analyzed for pesticides (*n* = 3,048) in the U.S. National Health and Nutrition Examination Survey (NHANES) 1999–2002 (CDC 2005a, 2005b). Pyrethroids are metabolized in mammals by ester hydrolysis and subsequent conjugation to a number of primary and secondary metabolites, including 3PBA (Godin et al. 2007). *In vitro* studies with human liver microsomes implicate carboxylesterases (Nishi et al. 2006; Ross et al. 2006), alcohol and aldehyde dehydrogenases (Choi et al. 2002), and cytochrome p450 (CYP450) isoforms (Godin et al. 2007) as responsible enzymes. A handful of studies using adult volunteers and pest control operators estimated half-lives of 3PBA in urine of 6–24 hr after pyrethroid exposure, with near complete elimination after several days (Eadsforth et al. 1988; Eadsforth and Baldwin 1983; Leng et al. 1996, 1997, 2003; Woollen et al. 1992).

Despite the NHANES evidence of widespread exposure in the U.S. population, little is known about how Americans are exposed to pyrethroids. Current research shows that both diet and nondiet factors may be important predictors of body burden. In their study of 23 children 3–11 of age in Seattle, Washington, Lu et al. (2006) found associations between urinary 3PBA and reported use of pyrethroids around the home and on pets, and eating conventional versus organic diets. Three small-scale (*n* < 200) studies conducted in 2002 by the CDC to evaluate exposures to pyrethroids sprayed for vector control found significant associations between urinary 3PBA and use of pesticides on pets in one study, but no significant difference in 3PBA before and after (1–4 days) spraying in any study (CDC 2005h). In their study of 386 mother–infant pairs in New York City, Berkowitz et al. (2003) found lower urinary 3PBA among women living in public versus private housing, but no consistent associations among 3PBA and other sociodemographic or pesticide use predictors. Williams et al. (2006) detected permethrin in plasma from 17 New York City women who reported using pyrethroids to control roaches at home versus none in samples from 21 women using alternative strategies.

Research conducted in Europe suggests that diet is an important exposure pathway. Urinary 3PBA has been regularly detected among German subjects reporting no household use or occupational exposure to pyrethroids (Heudorf and Angerer 2001; Heudorf et al. 2004; Schettgen et al. 2002). A small-scale study by Saieva et al. (2004) of 69 adult volunteers in two Italian cities provides similar indirect evidence for the influence of diet. In their national pilot study of German children 2–17 years of age (*n* = 396), Becker et al. (2006) found associations between urinary 3PBA and reported intake of boiled vegetables.

We used a regression approach to evaluate major diet and nondiet predictors of urinary 3PBA in the NHANES 1999–2002 pesticide subsample. We stratified subjects into child (6–10 years), teen (11–18 years), and adult (≥ 19 years) age groups based on the *a priori* assumption that exposure factors differ significantly by life stage (National Research Council 1993). Our objective was to explore the relative importance of diet versus nondiet predictors in explaining variability in urinary 3PBA for each age group. A secondary objective was to explore whether the NHANES data could be used to identify particular foods driving urinary 3PBA levels in each group. Our overall goal was to help identify major pathways of pyrethroid exposure in everyday American life and thus inform the design of future exposure and intervention studies.

## Methods

### NHANES data collection

Detailed data collection methods are available at the NHANES website (CDC 2007a, 2007b). NHANES was approved by the CDC Institutional Review Board. Briefly, urine samples were collected during the medical examination. Subjects were randomly stratified into morning (50% of subjects) and afternoon/evening sessions (50% of subjects), with morning sampling beginning at approximately 0830 hours, afternoon sampling at 1330 hours, and evening sampling at 1730 hours. Subjects in morning, afternoon, and evening sessions were requested to fast from 2300–0830 hours, 0730–1330 hours, and 1130–1730 hours, respectively (CDC 2001). Self-reported fasting duration was recorded before sample collection. A 24-hr dietary recall interview was conducted where subjects worked with trained interviewers using measurement aids to record all foods/beverages consumed from midnight to midnight the day before the examination (CDC 2005c). The recall data include estimates of grams eaten by descriptive eight-digit food code. To improve accuracy, the computer-assisted interview used in 1999–2001 was replaced by an automated multipass method in 2002 (CDC 2002). Although this method has been validated for assessing adult macronutrient intakes (Conway et al. 2004), we did not find peer-reviewed validation studies for children, teens, or consumption of individual foods.

Basic demographic data, as well as information on household pesticide use, physical activities, occupation, and other factors potentially relevant to pesticide exposures, were collected during the household sample person interview (CDC 2005d, 2005e). Eligible adult family members served as proxy respondents for subjects 6–15 of age. Information on use of pesticides in the home, yard, and foundation during the previous month was collected using a family questionnaire, administered with the subject or household head. Interviews were generally conducted 1 week before the examination, with the longest lag approximately 3 weeks (CDC 2005f). The pesticide use questions focused on chemicals used to “control fleas, roaches, ants, termites, or other insects” (CDC 2005d, 2005e).

We recoded the NHANES occupation data to identify subjects with potential work-place exposure. NHANES question OCD230 asked subjects the industry they worked in, and question OCD240 asked the type of work they performed. We created a new variable with five response categories—don’t work, other, farm, cleaning, and working in a private household—and categorized subjects based on their responses to questions OCD230 and OCD240.

### Exclusion/inclusion rules for regressions

We excluded subjects missing urinary 3PBA measurements, missing diet interview data, or with interviews coded “not reliable” (CDC 2005g). In the adult group, we also excluded pregnant subjects (18% of subjects tested) because urinary 3PBA levels differed significantly between pregnant and nonpregnant female subjects (Wilcoxon rank sum *p* = 0.0036).

Because the dietary recall period ended at midnight the night before sample collection, we included only subjects who attended the morning session and reported fasting ≥ 8 hr, hoping to exclude subjects who ate foods that were not recorded in the diet interview. We used 8 hr as the cutoff because that was the minimum duration between the midnight close of the recall period and the earliest hour of the morning examination session.

### Preparation of dietary interview data

Diet data for the pesticide subsample included 3,573 eight-digit descriptive food codes. Many (*n* = 1,031) of these foods were eaten by only one subject. In the teen and adult groups, we removed foods eaten by < 1% of the restricted sample, whereas we removed foods eaten by < 5% of the restricted child sample. Including these rarely eaten foods in the regressions would likely result in unstable parameter estimates and would not contribute to our understanding of variance in urinary 3PBA, so we decided to exclude them. We used 5% instead of 1% for the child group because the child sample size was approximately 30% and 16% of the teen and adult sample sizes, respectively.

### Regression model building

For each age group, we adapted initial model building from MacIntosh et al. (1997) and Romieu et al. (1990), involving the following steps:

#### Step 1

Construct a list of potential nondiet predictors of pyrethroid exposure from the literature (Appendix 1).

#### Step 2

Screen individual nondiet predictors by conducting univariate regressions of log 3PBA on individual nondiet predictors, passing only those with *p*-values ≤ 0.2 to step 3. The step 2 model form is


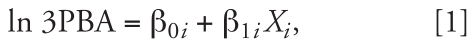


where ln 3PBA is the ln-transformed urinary 3PBA concentration; *i* indexes the step 1 nondiet predictors, (i.e., *i* = 1, *N*_ND_), and *N*_ND_ is the total number of nondiet predictors considered; *X_i_* represents the predictor in the univariate regression. These are class indicators for categorical variables or actual values for continuous variables.

#### Step 3

Perform a single Tobit regression (Tobin 1958) of 3PBA left-censored at the detection limit on all step 2 nondiet predictors, and select those predictors with *p*-value ≤ 0.2 for inclusion in the final model (predictors with *p*-values > 0.2 we manually removed from the step 3 regression until all remaining predictors had *p*-values ≤ 0.2). Tobit regression is appropriate for left-censored data sets with normally/lognormally distributed error terms (Helsel 1990; Kroll and Stedinger 1999; Lubin et al. 2004). The step 3 model form is


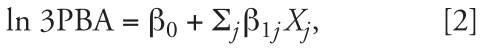


where *j* indexes the nondiet predictors (categorical and continuous) that pass the step 2 screen (note that this is a subset of the step 2 predictors, such that *j ≤ i* ); *X_j_* represents the predictor in the multivariable regression.

#### Step 4

Perform a backward elimination regression of the residuals from step 3 on reported 24-hr grams eaten by food code, and select only those food codes with *p*-values ≤ 0.2 for inclusion in the final model. The step 4 model form is


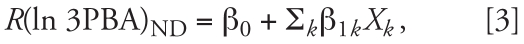


where *R*(ln 3PBA)_ND_ are the residuals from the step 3 regression, *k* indexes the individual food item (e.g., carrots), and *X_k_* indicates the number of grams of food item *k* consumed by each subject.

To control for urine dilution, we included creatinine as a predictor at each regression step. Creatinine is sometimes used this way because its excretion rate is independent of urine flow (Boeniger et al. 1993). Although this approach has limitations, including diurnal, seasonal, and age-related changes in creatinine excretion rates (Barr et al. 2005; Lu et al. 2006), urinary creatinine was the only physiologic variable available in NHANES for dilution correction.

### Final regression models

For each age group, we fitted the final model using Tobit regression, ln 3PBA, and only the predictors that passed steps 2–4. We included second-order polynomials and interaction terms for the nondiet predictors, whereas we tested food predictors for main effects only. The final model form is


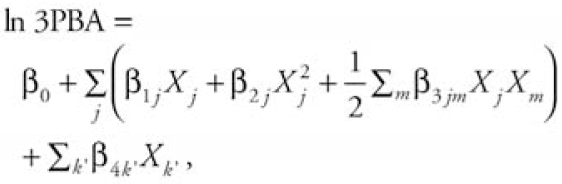


where β_0_ is the overall mean of the lntransformed urinary 3PBA concentration, *j* indexes the nondiet predictors, *m* indexes the nondiet predictor interaction terms, β_1_*_j_* is the linear regression coefficient for the *j*th nondiet predictor, β_2_*_j_* is the quadratic regression coefficient for the *j*th nondiet predictor, β_3_*_jm_* is the regression coefficient for the interaction between the *j*th and *m*th nondiet predictors, *k′* indexes the individual food predictor that passed step 4, β4*k′* is the linear regression coefficient for the *k′*th food predictor, and *X_k′_* indicates the number of grams of food *k′* consumed by each subject.

After step 3, step 4, and the final model fitting, we evaluated our model assumptions (normality, homoskedasticity) by examining plots of predicted values versus standardized residuals, and histograms and normal probability plots of standardized residuals. We used PROC LIFEREG and PROC REG in SAS 9.1 (SAS Institute Inc. 2004) for the Tobit and backward elimination regressions, respectively. We specified a lognormal distribution for the Tobit regressions, and left 3PBA values censored at the reported detection limit (0.07 mg/L) (CDC 2005a, 2005b). Our criterion for statistical significance was *p ≤* 0.05 for the regression parameters.

The NHANES data include sample weights and other design variables ordinarily used to adjust statistical inferences to represent the U.S. population. We chose not to apply these weights or other design adjustments because we applied numerous exclusion criteria. Further, we were concerned with exploring relationships among urinary 3PBA and diet/nondiet exposure factors in a convenient sample rather than generalizing results to the U.S. population.

### Bootstrap simulations

We conducted bootstrap simulations (Efron 1979) to evaluate the impact of the model selection procedure on variability in the regression parameters for the food predictors. Ordinary confidence intervals and *p*-values account for the effects of sample size given a particular model, but the model selection procedure adds additional variability. Because the consumption data for each food predictor were typically characterized by a large number of zero values, and a smaller number of nonzero values depending on the number of subjects who ate that food, the regression parameters may be unduly influenced by outlying points. The bootstrap approach allows evaluation of whether certain foods remain significant predictors if we randomly remove observations, thus testing the influence of potentially outlying points. We hypothesized that foods appearing as significant predictors in most of the simulated regressions would have a significant association with urinary 3PBA that was not simply due to chance.

**Figure 1:**
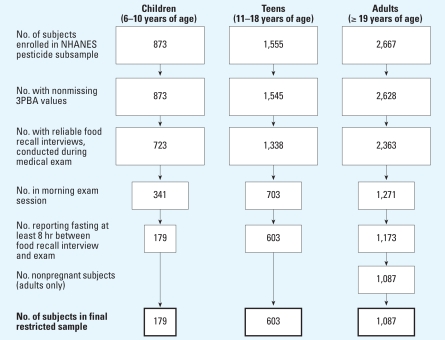
Sample restriction and final sample sizes. Box areas represent proportion of restricted group to original number of subjects enrolled in NHANES pesticide sample, by age group.

To create each bootstrapped sample, we used PROC SURVEYSELECT in SAS 9.1 to sample *n* random observations with replacement from the restricted data set, where *n* is the restricted sample size (Figure 1). We then performed the regression procedures described above on the bootstrapped sample, with one exception: because we automated the bootstrap simulations, we did not manually remove predictors with *p*-values > 0.20 during step 3. If ≥ 5% of subjects in the child group or 1% in the teen and adult groups did not eat a particular food in the bootstrapped sample, we did not include that food in the step 4 regression. We repeated this process 5,000 times for each age group. We constructed box plots of the bootstrapped regression parameters for food codes that were significant predictors of urinary 3PBA in the original regressions.

## Results

### Restricted sample sizes

Figure 1 shows the final restricted sample sizes by age group and the number of subjects removed by each exclusion rule. Final sample sizes for the child, teen, and adult groups were 179, 603, and 1,087, respectively. The number of incomplete observations (i.e., missing data for one or more predictors) did not exceed 2% of total observations for any age group. Final sample sizes were approximately 40% of the starting sample size, with the exception of the child group, for which we excluded an additional 20% of subjects for not meeting the ≥8-hr fasting criterion.

Among the restricted samples, we found no significant difference in 3PBA detection frequencies by age group (Fisher’s exact *p* = 0.1623). However, creatinine-adjusted urinary 3PBA differed significantly (Wilcoxon rank sum *p* < 0.0001), with median/95th percentile values of 0.4/4.8, 0.2/1.6, and 0.3/3.1 ng/mg creatinine for the child, teen, and adult groups, respectively. We substituted the detection limit for below-detection values for all analyses reported in this section. We did not normalize urinary 3PBA by creatinine in the regressions.

### Model specifications and fit diagnostics

The screening steps reduced the number of predictors from 68 (16 nondiet/52 diet) to 17 (1 nondiet/16 diet) in the child group, from 278 (27 nondiet/251 diet) to 84 (5 nondiet/79 diet) in the teen group, and from 296 (27 nondiet/269 diet) to 93 (15 nondiet/78 diet) in the adult group. Probability plots of standardized residuals supported the lognormality assumptions, whereas plots of standardized residuals versus predicted 3PBA values showed even scatter across the range of predicted values for each group. We evaluated model fit by comparing the difference between –2 log likelihood for the final fitted model and that of a reduced model containing the same observations but only urinary creatinine as a predictor (creatinine-only model) (Collett 1994). We tested two other models using the same observations—one with only nondiet predictors, the other with only food predictors—against the creatinine-only model for each age group. For all groups, all models tested explained significantly more of the variability in urinary 3PBA than did the creatinine-only model (i.e., chi-square *p*-values for all tests were < 0.05).

### Significant predictors of urinary 3PBA by age group

Tables 1 and 2 show the regression parameter estimates and corresponding *p*-values for significant predictors of urinary 3PBA in the child and teen, and adult groups, respectively. Urinary creatinine (milligrams per deciliter) was a significant predictor (*p* < 0.0001) of urinary 3PBA in all three groups. Eight predictors (including creatinine and the intercept) were significant in the child model; significant foods included ground beef, toasted white bread, ice cream, tortilla chips, cheese, and cookies. In the teen model, significant predictors of urinary 3PBA included body mass index (BMI), BMI^2^, and 21 foods. In the adult model, being an active tobacco user was significantly associated with urinary 3PBA, as were two occupation categories (other vs. don’t work, and private household vs. don’t work). Last, 15 foods were significant predictors of urinary 3PBA in the adult model.

### Bootstrap results by age group

**Figure 2:**
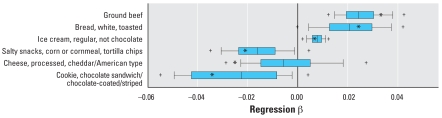
Distributions of regression parameters (β) from 5,000 bootstrap simulations using the restricted child data set and original Tobit regression approach (only significant food predictors from the main regression are shown). Box width indicates 25th to 75th percentiles, with median shown by the central line; whiskers mark 10th and 90th percentiles; and plus symbols mark 5th and 95th percentiles. Asterisks mark β from regression using the original data set.

**Figure 3:**
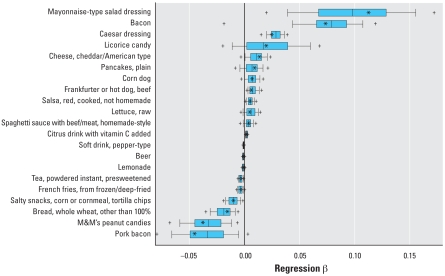
Distributions of regression parameters (β) from 5,000 bootstrap simulations using the restricted teen data set and original Tobit regression approach (only significant food predictors from the main regression are shown). Box width indicates 25th to 75th percentiles, with median shown by the central line; whiskers mark 10th and 90th percentiles; and plus symbols mark 5th and 95th percentiles. Asterisks indicate β from regression using the original data set.

**Figure 4:**
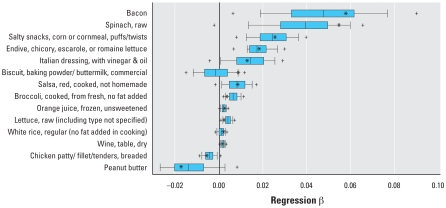
Distributions of regression parameters (β) from 5,000 bootstrap simulations using the restricted adult data set and original Tobit regression approach (only significant food predictors from the main regression are shown). Box width indicates 25th to 75th percentiles, with median shown by the central line; whiskers mark 10th and 90th percentiles; and plus symbols mark 5th and 95th percentiles. Asterisks indicate β from regression using the original data set.

Tables 1 and 2 also present results from the bootstrap analyses including the percentage of time that certain predictors appeared in the bootstrap sample (a bootstrap “hit”), the percentage of hits that passed the food screen, and the percentage of hits significant in the final model. Figures 2–4 present box plots of the bootstrapped regression parameters (β) for significant foods in the child, teen, and adult regressions, respectively. In the child group, only ground beef had a 5th–95th percentile bootstrapped β range above zero (Figure 2). In the teen group, mayonnaise-type salad dressing and Caesar dressing had 5th–95th percentile ranges above zero, whereas pepper-type soft drinks, presweetened instant tea, tortilla chips, whole wheat bread, and M&Ms peanut candies had 5th–95th percentile ranges below zero (Figure 3). In the adult group, foods with 5th–95th percentile bootstrapped β ranges above zero included bacon, corn puffs/twists, and endive/chicory/escarole/romaine lettuce; no adult foods had 5th–95th percentile ranges below zero.

## Discussion

### Statistical significance versus random chance

Because we compared a large number of predictors with urinary 3PBA levels, it was reasonable to expect that spurious associations may be found due to chance. For example, in a case where no predictors were associated with a response variable when we compared 100 predictors with the response variable using a 5% cutoff for probability, approximately five predictors would be expected to display statistical significance due to chance. If we found a larger number of predictors (i.e., > 5%), we would be confident that at least some of the predictors represented true association rather random chance. Following this example, in the child, teen, and adult regressions, we would expect 1, 5, and 5 predictors, respectively, to be significant due to chance alone. However, 8 predictors were significant in the child model, 23 in the teen model, and 21 in the adult model. This suggests that at least some are indeed predictors of urinary 3PBA in these groups and that the associations we see are not all due purely to chance.

### Pesticide use predictors of urinary 3PBA

Diet and household pesticide use are hypothesized to be major contributors to pyrethroid body burden in non-occupationally exposed Americans. In our analyses, diet was a significant predictor of urinary 3PBA for all three age groups. This was not unexpected because one or more pyrethroids were detected in 52% of the 260 food items analyzed for pesticides in the 1999–2001 Total Diet Study by the U.S. Food and Drug Administration (FDA 2001).

Reported household pesticide use was not a significant predictor of urinary 3PBA in any age group. A possible methodologic explanation for the observed lack of association in the child and teen models may lie in the fact that the NHANES interviews were conducted with proxy respondents for subjects < 16 years of age, whereas subjects ≥ 16 years of age answered themselves (CDC 2001). The lack of association could be due to true differences in pyrethroid use practices and behaviors in children and teens versus adults. For example, parents may store pesticides out of reach or prohibit their children or teens from using them. Parents of young children may use fewer pesticides at home. Although we did not observe a difference in reported indoor use among the three groups, a greater percentage of adult subjects reported using pesticides in the yard (16% vs. 9% and 14% in the child and teen groups, respectively). Likewise, the percentage of teen and adult subjects reporting nonprofessional yard applications (10–11%) was twice that reporting professional applications (4–6%), whereas similar percentages reported nonprofessional and professional applications in the child group (4–5%). This may reflect underreporting of yard pesticide use by adult proxy respondents of child subjects.

The lack of association between reported household pesticide use and urinary 3PBA in our child and teen models contradicts the findings of Lu et al. (2006), although study design differences limit comparison. Lu et al. collected 15 consecutive days of urine samples and asked about household pesticide use during the past month. They also administered their questionnaire on sampling day 1, whereas the NHANES household pesticide use questionnaire was administered 1–3 weeks before sampling. The narrower window of time between potential exposure and urine collection in the Lu et al. (2006) study may partly explain the contradictory findings. The mismatch in ages between Lu et al. (3–11 years of age) and the restricted NHANES sample (6–10 years of age) may also help explain the difference because younger children have been shown to exhibit greater hand-to-mouth activity than do older children (Xue et al. 2007), potentially resulting in higher contaminant body burdens. It is important to note that Lu et al. (2006) studied a small cohort of metropolitan Seattle, Washington (USA) families, whose pesticide use practices may not be representative of other U.S. regions.

In their study of German children, Becker et al. (2006) found significant associations between reported indoor pesticide use (yes/no) and urinary levels of other pyrethroid metabolites but not 3PBA. They did find a significant association between 3PBA and permethrin concentrations in house dust, which might be argued is a more accurate measure of indoor use than is self-reported use during the previous month because the half-life of pyrethroids in dust may be longer than 1 month (Leng et al. 2005). Nonetheless, Becker et al. cite three other German studies, across a range of subject ages, in which the dust–3PBA relationship was not significant.

### Other significant nondiet predictors of urinary 3PBA

Significant nondiet predictors in the teen model included urinary creatinine and BMI and their square terms, indicating possible nonlinear associations between these predictors and 3PBA. Creatinine and its square term were also significant in the adult model, as was active tobacco use. We originally included tobacco use as a predictor because of its status as a known CYP450 inducer (Appendix 1). However, we cannot exclude the possibilities of direct exposures from tobacco (although most pyrethroids currently are not registered for use on U.S. tobacco) or indirect exposures to pyrethroid-contaminated dust from increased hand-to-mouth activity potentially associated with tobacco use.

The occupation variable did not return the categories we expected to be significant, namely, farm (NHANES categories farm operators/managers/supervisors, farm/nursery workers, and related agricultural/forestry/fishing occupations) and cleaning (NHANES cleaning/building service occupations category) (CDC 2005i). Instead, working in a private household or other jobs (a composite of all other NHANES categories not coded farm, cleaning, or household) were significantly associated with lower 3PBA versus not working (Table 2).

In the adult model, two nondiet predictors had *p*-values close to our significance criterion: reported number of prescription medications known to inhibit CYP450 used in the past month (β = 1.7 ×10^−1^; *p* = 0.0652), and minutes spent gardening in the past month (β = 5.3 × 10^−1^; *p* = 0.0613). In the restricted adult sample, 14%, 3%, and 1% of subjects reported taking one, two, or three CYP450-inhibiting prescription drugs, respectively; 41 drugs inhibiting a range of CYP450 isoforms were reported. Di Consiglio et al. (2005) demonstrated with human *in vitro* preparations that several organophosphate pesticides inhibit CYP450-mediated degradation of an antidepressant medication, but we were unable to find studies on the effect of therapeutic drugs on pesticide metabolism in general, or pyrethroids specifically. The question of drug influences on pesticide pharmacokinetics may be worthy of further exploration. Because only three of the subjects who reported gardening in the past month also reported using pesticides, we did not expect to see any relationship between gardening activity and urinary 3PBA.

### Specific foods driving urinary 3PBA levels by age group

For all age groups, the regressions produced a greater number of foods that were significantly associated with urinary 3PBA than might have been expected due to chance. Further interpretation is complicated by the fact that the regressions returned foods with both positive and negative β values. We find this difficult to explain beyond the simple argument we outlined in a similar study (Ryan et al. 2001), namely, that a positive β suggests that the corresponding food item contained pyrethroids whereas a negative β suggests the corresponding food did not contain pyrethroids and substituted for one that did.

In the present study, we used simple bootstrap resampling to provide additional insight into how variance in the NHANES food data might influence the β estimates. Certain foods remained significant in a majority (which we define arbitrarily as 70% or more) of the bootstrap simulations, suggesting that the data variability did not affect the contribution of these particular foods in explaining the variance in urinary 3PBA. These foods were not always the most frequently consumed, for example, Caesar dressing in the teen group. Foods that were significant in 70% or more of the simulations and had 5–95th percentile bootstrap β ranges above zero included ground beef in the child group and mayonnaise-type salad dressing, bacon, and Caesar dressing in the teen group. No foods met the 70% criterion with 5–95th percentile β ranges below zero.

### Limitations and future research

We did not apply NHANES sample weights or other design variables during our analyses, preventing us from generalizing our results to the U.S. population. The inclusion of the NHANES design variables is unlikely to change the regression coefficients substantially because our models controlled or stratified by age and race, two primary characteristics used for oversampling in NHANES. However, incorporation of the design variables could affect the standard errors and statistical significance of the predictors (Horton and Fitzmaurice 2004).

Our work demonstrates the limitations of using large observational data sets like NHANES for evaluating exposure–biomarker relationships. We chose an exploratory approach because the current literature on pyrethroid exposures and urinary 3PBA is inconsistent, resulting in many reasonable *a priori* hypotheses. From this starting point, we used a multistep model selection procedure to screen predictors based on *p*-values. The dependence of *p*-values on sample size is well known, and the large sample sizes especially in our adult group may have resulted in a failure to screen out certain foods that were truly unrelated to 3PBA. We used a bootstrap approach to evaluate the impact of this model selection procedure on variability in the regression parameters for the food predictors. The bootstrap results presented in Tables 1 and 2 demonstrate how certain foods, even those eaten relatively frequently in the original sample (e.g., lettuce), appear as significant predictors only in a fraction (e.g., lettuce = 42%) of the bootstrapped regressions, whereas Figures 2–4 demonstrate that the estimated β values for many foods are not significantly different from zero after a large number of simulations. This illustrates one potential pitfall associated with drawing conclusions from a single regression model of a large data set with many low-frequency predictors: slight permutations of the data such as those achieved by random resampling may lead to different conclusions.

Another limitation is that we did not test interactions between certain foods likely to have been eaten together (e.g., salad dressing and lettuce) because we did not have adequate degrees of freedom. Failure to account for these correlations may produce unstable regression coefficients that are influenced by outlying points and may not be readily interpretable for certain combinations of foods due to the arbitrary partitioning of variance between them. Additional research is needed on which foods are most often eaten together and which foods are likely to contain pyrethroid residues. Further, we did not distinguish between foods eaten at home versus out, although NHANES provided this information; this could have biased our results if more pyrethroids are typically present at one of these locations. Last, we cannot rule out the possibility that there were important predictors of urinary 3PBA that we did not consider simply because they were not included in NHANES.

Despite these limitations, our results point toward one practical conclusion and several interesting hypotheses for future testing. The practical conclusion is that we did not detect an association between reported household pesticide use and urinary 3PBA in the NHANES data. Future survey directors may consider collecting more detailed information about pesticide use events, including dates, chemical names, and/or application types, although this would add to subject burden. If the goal of NHANES is to provide cross-sectional reference values, and not to link exposure factors with biomarker levels, then the cost of a more detailed pesticide use questionnaire may not be justified.

Future hypotheses to be tested include the association between urinary 3PBA and consumption of the specific foods with positive β values in our bootstrap analyses (e.g., ground beef in the child model). This might be accomplished with the current NHANES data by aggregating the eight-digit food codes into larger categories (e.g., by grouping all the bacon codes). However, these associations might be more efficiently investigated using diet and 3PBA data from ongoing longitudinal studies (e.g., Lu et al. 2006). Our results are also suggestive of potential interactions between prescription medications and pyrethroid metabolism, but research characterizing specific metabolic and interaction pathways in humans is needed. Interactions between pharmaceuticals and dietary pesticide metabolism might be investigated through controlled studies where matched subjects eat the same foods and differ only by medication status.

## Correction

In the manuscript originally published online, data were missing from Figure 3; the figure has been corrected here.

## Figures and Tables

**Table 1:** Significant predictors of urinary 3PBA in NHANES 1999–2002 and results of bootstrap analyses for children and teens.

Predictor	Main regression		Percent doers/eaters in original data	Percent time in bootstrap sample (“hit”)	Percent hits with significant *p*-values	
	Parameter estimates	*p*-Value			After food screen (*p ≤* 0.2)	After final regression (*p ≤* 0.05)
Children (6–10 years of age) (restricted sample, *n* = 179; foods eaten by at least 5% of subjects)						
Ground beef (g)	3.3 ×10^−2^	< 0.0001	6	21	96	93
Bread, white, toasted (g)	2.4 ×10^−2^	0.0332	7	44	59	30
Urinary creatinine (mg/dL)	1.3 ×10^−2^	< 0.0001	100	100	NA	99
Ice cream, regular, not chocolate (g)	6.6 ×10^−3^	0.0288	5	6	68	55
Salty snacks, corn, tortilla chips (g)	−2.1 ×10^−2^	0.0158	13	99	40	19
Cheese, processed, cheddar/American (g)	−2.5 ×10^−2^	0.0154	13	99	50	10
Cookie, chocolate sandwich/coated/striped (g)	−3.4 ×10^−2^	0.0046	5	6	60	29
Intercept	−2.2 ×10^0^	< 0.0001	NA	100	NA	100
Teens (11–18 years of age; restricted sample, *n* = 603; foods eaten by at least 1% of subjects)						
Mayonnaise-type salad dressing (g)	1.1 ×10^−1^	< 0.0001	3	100	86	72
Bacon (meat type not specified) (g)	7.4 ×10^−2^	0.0003	1	70	84	73
Caesar dressing (g)	2.5 ×10^−2^	< 0.0001	1	69	93	89
Licorice candy (g)	2.0 ×10^−2^	0.0409	1	89	87	45
Urinary creatinine (mg/dL)	1.7 ×10^−2^	< 0.0001	100	100	NA	100
Cheese, cheddar/American type (g)	1.4 ×10^−2^	0.0051	5	100	80	44
Pancakes, plain (g)	9.3 ×10^−3^	0.0227	2	98	54	23
Corn dog (g)	6.9 ×10^−3^	0.0177	2	94	61	32
Frankfurter or hot dog, beef (g)	6.3 ×10^−3^	0.0036	2	99	76	63
Salsa, red, cooked, not homemade (g)	5.0 ×10^−3^	0.0020	6	100	68	51
Lettuce, raw (g)	4.9 ×10^−3^	0.0334	21	100	60	36
Spaghetti sauce with meat, home style (g)	3.6 ×10^−3^	0.0463	1	70	60	34
BMI^2^ [(kg/m^2^)^2^]	3.0 ×10^−3^	0.0038	100	100	NA	70
Citrus drink with vitamin C added (g)	1.9 ×10^−3^	0.0006	2	98	80	71
Urinary creatinine^2^ [(mg/dL)^2^]	−2.6 ×10^−5^	< 0.0001	100	100	NA	100
Soft drink, pepper-type (g)	−7.0 ×10^−4^	0.0158	8	100	91	64
Beer (g)	−8.0 ×10^−4^	0.0320	1	55	69	49
Lemonade (g)	−1.4 ×10^−3^	0.0054	3	100	66	37
Tea, instant, presweetened with sugar (g)	−2.7 ×10^−3^	0.0287	1	70	59	46
French fries, from frozen/deep fried (g)	−3.2 ×10^−3^	0.0122	18	100	72	48
Salty snacks, corn, tortilla chips (g)	−9.5 ×10^−3^	0.0018	13	100	78	61
Bread, whole wheat, other than 100% (g)	−1.5 ×10^−2^	0.0004	4	100	59	53
M&M’s peanut candies (g)	−3.8 ×10^−2^	0.0002	1	89	80	62
Pork bacon (g)	−4.5 ×10^−2^	0.0172	2	100	80	36
BMI (kg/m^2^)	−1.8 ×10^−1^	0.0014	100	100	NA	75

NA, not applicable.

**Table 2:** Significant predictors of urinary 3PBA in NHANES 1999–2002 and results of bootstrap analysis for adults (restricted sample, *n* = 1,087; foods eaten by at least 1% of subjects).

Predictor	Main regression		Percent doers/eaters in original data	Percent time in bootstrap sample (“hit”)	Percent hits with significant *p*-values	
	Parameter estimate	*p*-Value			After food screen (*p ≤* 0.2)	After final regression (*p ≤* 0.05)
Active tobacco user	2.3 ×10^−1^	0.0326	26	100	NA	49
Bacon (g)	5.8 ×10^−2^	0.0053	2	97	48	31
Spinach, raw (g)	5.5 ×10^−2^	< 0.0001	1	74	61	48
Salty snacks, corn or cornmeal, puffs/twists (g)	2.6 ×10^−2^	0.0094	2	97	71	55
Urinary creatinine (mg/dL)	1.6 ×10^−2^	< 0.0001	100	100	NA	99
Endive, chicory, escarole, or romaine lettuce (g)	1.8 ×10^−2^	< 0.0001	3	100	79	68
Italian dressing, with vinegar and oil (g)	1.3 ×10^−2^	0.0270	4	100	49	31
Biscuit, baking powder/buttermilk, commercial (g)	8.9 ×10^−3^	0.0197	2	100	30	6
Salsa, red, cooked, not homemade (g)	8.6 ×10^−3^	0.0097	5	100	72	46
Broccoli, cooked, from fresh, no fat added (g)	3.7 ×10^−3^	0.0461	2	100	58	47
Orange juice, frozen, unsweetened (g)	2.8 ×10^−3^	0.0076	2	95	86	58
Lettuce, raw (including type not specified) (g)	2.5 ×10^−3^	0.0423	25	100	64	53
White rice, regular (no fat added in cooking) (g)	2.2 ×10^−3^	0.0133	5	100	40	14
Wine, table, dry (g)	1.6 ×10^−3^	0.0089	4	100	52	42
Urinary creatinine^2^ [(mg/dL)^2^]	−1.9 ×10^−5^	< 0.0001	100	100	NA	100
Chicken patty/fillet/tenders, breaded (g)	−5.5 ×10^−3^	0.0149	2	99	56	32
Peanut butter (g)	−1.7 ×10^−2^	0.0159	4	100	38	19
Occupation (other vs. don’t work)	3.8 ×10^−1^	0.0004	59	100	NA	47
Occupation (private household vs. don’t work)	−1.4 ×10^0^	0.0440	1	100	NA	65
Intercept (ln 3PBA)	−4.3 ×10^0^	0.0028	NA	100	NA	26

NA, not applicable.

## Appendix

**Appendix 1.** NHANES predictor variables (categories/units) used in 3PBA regression analyses (all variables self-reported except BMI and urinary DEET)

Diet
Previous 24-hr reported grams consumed by NHANES eight-digit food code (grams, including 0)
Total minutes fasted before NHANES medical exam (minutes)
Nondiet
Demographics
Sex (female/male)
Race/ethnicity (Mexican American/other Hispanic/non-Hispanic white/non-Hispanic black/other race)
BMI [weight (kg)/height (m)^2^]
Occupation (adult only) [recoded as don’t work, other, farm (including farm, nursery, and related agricultural occupations), cleaning (including cleaning and building service occupations), working in a private household]
Household pesticide use
Pest control in home in past month (yes/no)
Rooms treated for pests (no rooms/entire household/kitchen or dining room/other rooms)
No. of treatments in home by nonprofessional (0 times/1 time/2 or more times)
No. of treatments in home by professional (0 times/1 time/2 or more times)
No. of treatments in yard by nonprofessional [0 times (including no yard)/1 time/2 or more times]
No. of treatments in yard by professional [0 times (including no yard)/1 time/2 or more times]
Foundation/outside of building treated (yes/no)
Urinary DEET (detected/nondetected)
Physical activity
Rigorous tasks around home/yard past 30 days (e.g., heavy cleaning; teen/adult only) (yes/no)
Frequency of tasks around home/yard past 30 days (number, including 0)
How long each time (minutes, including 0)
Gardening past 30 days (teen/adult only) (yes/no)
No. of times gardening past 30 days (number, including 0)
Minutes gardening past 30 days (including 0)
Yard work in past 30 days (teen, adult only) (yes/no)
No. of times yard work past 30 days (number, including 0)
Minutes yard work past 30 days (including 0)
Children’s games in past 30 days (child only) (yes/no)
No. of times children’s games past 30 days (number, including 0)
Minutes children’s games past 30 days (including 0)
Intake of CYP450 inhibitors^a^
Reported past month use of ≥1 prescription medications known to inhibit CYP450 (yes/no, no response)
Reported number of CYP450-inhibiting prescription medications used past month (number, including 0)
Intake of CYP450 inducers^a^
Reported past month use of ≥1 prescription medications known to induce CYP450 (yes/no, no response)
Used tobacco or nicotine last 5 days (teen/adult only) (yes/no)
Active tobacco user (teen/adult only) (yes/no)
Recent (i.e., past 24 hr) tobacco exposure (serum cotinine detected/not detected)

Abbreviations: CYP450, cytochrome P450; DEET, *N,N*-diethyl-*meta*-toluamide.

a Data from human CYP450 inhibitors/inducers (Flockhart 2005).
